# Precision computerised cognitive behavioural therapy (cCBT) for adolescents with depression: a pilot and feasibility randomised controlled trial protocol for SPARX-UK

**DOI:** 10.1186/s40814-024-01475-7

**Published:** 2024-03-26

**Authors:** K. Khan, C. L. Hall, C. Babbage, S. Dodzo, C. Greenhalgh, M. Lucassen, S. Merry, K. Sayal, K. Sprange, K. Stasiak, C. R. Tench, E. Townsend, P. Stallard, C. Hollis, on behalf of the Digital Youth research team

**Affiliations:** 1 Mental Health & Clinical Neurosciences, School of Medicine, University of Nottingham, Nottingham, UK; 2 NIHR MindTech MedTech Co-operative, Institute of Mental Health, University of Nottingham, Nottingham, NG7 2TU UK; 3 NIHR Nottingham Biomedical Research Centre, Nottingham, UK; 4 School of Computer Science, University of Nottingham, Nottingham, UK; 5 School of Health and Psychological Sciences, University of London, London, UK; 6 School of Medicine, University of Auckland, Auckland, New Zealand; 7 Centre for Mood Disorders, Institute of Mental Health, University of Nottingham, Nottingham, UK; 8 Nottingham Clinical Trials Unit, University of Nottingham, Nottingham, UK; 9 Precision Imaging Beacon, Queen’s Medical Centre, Nottingham, UK; 10 School of Psychology, University of Nottingham, Nottingham, UK; 11 Department for Health, University of Bath, Bath, UK

**Keywords:** Serious game, CBT, Complex intervention, Adolescents, Digital intervention, Depression

## Abstract

**Background:**

A serious game called SPARX (Smart, Positive, Active, Realistic, X-factor thoughts), originally developed in New Zealand and incorporating cognitive behavioural therapy (CBT) principles, has been shown to help reduce symptoms of depression and anxiety in adolescents with mild to moderate depression in studies undertaken in Australasia. However, SPARX has never been trialled in the United Kingdom (UK), and there have been issues relating to low engagement when it has been used in a real-world context.

**Aims:**

To conduct the first pilot and feasibility randomised controlled trial (RCT) in England to explore the use of SPARX in different settings. The trial will explore whether SPARX supported by an e-coach (assistant psychologists) improves adherence and engagement compared with self-directed (i.e. self-help) use. The trial results will be used to inform the optimal mode of delivery (SPARX supported vs. SPARX self-directed), to calculate an appropriate sample size for a full RCT, and to decide which setting is most suitable.

**Methods:**

Following consultation with young people to ensure study suitability/appropriateness, a total of 120 adolescents (11–19 years) will be recruited for this three-arm study. Adolescents recruited for the study across England will be randomised to receive either SPARX with human support (from an e-coach), self-directed SPARX, or a waitlist control group. Assessments will be conducted online at baseline, week 4, and 8–10-week post-randomisation. The assessments will include measures which capture demographic, depression (Patient Health Questionnaire modified for adolescents [PHQ-A]) and anxiety (Revised Child Anxiety and Depression Scale [RCADS]) symptomatology, and health-related quality-of-life data (EQ-5D-Y and proxy version). Analyses will be primarily descriptive. Qualitative interviews will be undertaken with a proportion of the participants and clinical staff as part of a process evaluation, and the qualitative data gathered will be thematically analysed. Finally, feasibility data will be collected on recruitment details, overall study uptake and engagement with SPARX, participant retention, and youth-reported acceptability of the intervention.

**Discussion:**

The findings will inform the design of a future definitive RCT of SPARX in the UK. If the subsequent definitive RCT demonstrates that SPARX is effective, then an online serious game utilising CBT principles ultimately has the potential to improve the provision of care within the UK’s health services if delivered en masse.

**Trial registration:**

ISRCTN: ISRCTN15124804. Registered on 16 January 2023, https://www.isrctn.com/ISRCTN15124804.

**Supplementary Information:**

The online version contains supplementary material available at 10.1186/s40814-024-01475-7.

## Key messages regarding feasibility

- What uncertainties exist regarding the feasibility?
  The feasibility of using an online serious game intervention (SPARX) for adolescents with mild to moderate depression is currently unknown in the United Kingdom (UK). Furthermore, the viability of utilising this intervention in different settings, and whether there is an added benefit of limited human support on engagement rates, is unknown, and, in particular, it is important to assess the impact on recruitment rates, retention rates, study completion rates, acceptability of the intervention, and scalability to power a subsequent RCT.

## Background

### Adolescent depression

Depression amongst adolescents and young people has become a pressing public health issue. Global prevalence rates show that elevated self-reported depressive symptoms for adolescents were 34%, and the prevalence for major depressive disorder and dysthymia was 8% and 4%, respectively [1], with increased rates reported as a result of the COVID-19 pandemic [2]. Depression has serious adverse effects on social, academic, and family functioning [3], with early onset depression being associated with higher rates of suicide attempts and suicide compared to individuals with no psychiatric disorders [4, 5]. Notably, suicide is the fourth leading cause of death in young people globally [6]. The overall impact of untreated depression can be devastating for an adolescent and their family as well as costly to society. Early onset mental health problems are estimated to cost United Kingdom (UK) society £70–100 billion per year [7]. Therefore, it is imperative that adolescents are given timely treatment for their symptoms, not only for their mental health and outcomes but also to benefit the socioeconomic welfare of society more generally.

### Barriers to treatment

There are effective interventions available for adolescent depression with current National Institute for Health and Care Excellence (NICE) guidelines recommending cognitive behavioural therapy (CBT) as a first-line treatment [8]. However, access to evidence-based treatment such as CBT is low amongst all demographics [9] with only 25% of adolescents in the UK receiving appropriate treatments [10]. Studies have noted considerable barriers for adolescents accessing care [11]. Due to their affinity with technology, promising developments that may benefit some adolescents are online or digital health interventions (DHIs). One effective approach for adolescents with depression is computerised CBT (cCBT), with numerous randomised controlled trials (RCTs) and meta-analyses demonstrating their efficacy [12, 13]. As a result, cCBT is recommended by NICE as part of a stepped-care model for the management of adolescents with mild depression [14]. With progress in digitised technology, cCBT has become more interactive and aesthetically attractive to adolescents, particularly with the advent of ‘serious games’ [15]. The idea behind “serious games” (and gamification more generally) is for there to be a primary purpose other than pure entertainment (e.g. learning and behaviour change) [16]. The hope is that serious games (or adding gaming elements to an intervention) will make cCBT more engaging and user friendly whilst addressing important health issues.

### The evidence for SPARX

One serious CBT game is called SPARX (Smart, Positive, Active, Realistic, X-factor thoughts). Originally developed in New Zealand [17], where SPARX is publicly available, it uses CBT principles and techniques to help address symptoms of depression and anxiety in adolescents with mild to moderate depression. SPARX is designed as a self-help intervention where the user navigates their own way through a virtual universe, developing CBT skills as they progress. This involves participants undertaking a series of challenges to restore the balance in a fantasy world dominated by GNATs (Gloomy Negative Automatic Thoughts). The first RCT using SPARX was a non-inferiority trial in New Zealand where SPARX was compared with treatment as usual (TAU) amongst adolescents seeking help for their depression [17]. Per protocol analyses showed that SPARX was not inferior to TAU (face-to-face therapy) with a post-intervention mean reduction on the primary outcome measure (Children’s Depression Rating Scale-Revised) of 10.32 for SPARX compared to 7.59 for TAU. Improvements were maintained at 3-month follow-up. It was also reported to be acceptable and safe.

Subsequent trials of SPARX have been conducted with adolescents with depression in New Zealand. For instance, Fleming et al. [18] conducted a pragmatic RCT and found there were significantly greater reductions in depression and anxiety symptoms from baseline to week 5 for the SPARX group compared with a wait-list control, with gains being maintained at 10-week follow-up amongst young people excluded from mainstream schools. Lucassen et al. [19] found a significant decrease in depression and anxiety symptoms in sexual minority (e.g. lesbian, gay and bisexual) adolescents using the Rainbow version of SPARX from pre- to post-intervention, which were maintained at follow-up. Most sexual minority participants said they would recommend the adapted version of SPARX to a friend (80%) and thought it would appeal to other young people (85%). More than 90% of participants reported completing four or more modules of Rainbow SPARX (mean 6.6 modules, range 1–7). Seventeen participants (81%) reported finishing all seven modules, thus suggesting that the adapted Rainbow SPARX intervention was acceptable and could be feasibly delivered. Finally, a stepwise cohort study design by Fleming et al. [20] used an adapted resilience version of SPARX in a youth offenders’ programme, but the sample was too small (*n* = 19) to evaluate the intervention as planned. Due to the limited engagement with SPARX, and the largely neutral or negative feedback from youth and social workers, the authors felt that the adapted resilience version of SPARX was not shown to be acceptable or feasible with this service user group in this setting.

Three trials using SPARX have been completed outside of New Zealand to date. One RCT used SPARX as a depression prevention tool and was conducted in the Netherlands [21]. This showed no difference between SPARX and other conditions including group-based CBT offered in person (and most notably a monitoring control). Similarly, a small cluster RCT conducted in Ireland found no significant effects compared with a no intervention control [20]. The most recent trial was a depression prevention study carried out in Australia [22]. This cluster RCT found that participants in the adapted resilience version of SPARX condition showed significantly reduced depression symptoms on the Major Depression Inventory relative to the control group at post-intervention (*d* = 0.29) and 6-month post-baseline (*d* = 0.21) but not at 18-month post-baseline (*d* = 0.33).

Taken together, the evidence for SPARX is primarily restricted to studies undertaken in Australasia. Evaluations conducted elsewhere have not found clear benefits of SPARX compared to no-treatment controls [20, 21]. Therefore, we cannot assume that the evidence for SPARX internationally has been established. Digital interventions are context dependent, and positive findings in one nation may not translate to other countries [23]. There is a need to evaluate SPARX outside of its region of origin, and the present study is the first UK trial to do this.

### The issue of engagement with DHIs

Whilst DHIs for adolescents such as SPARX have shown encouraging outcomes, there have been several issues relating to low engagement, which in the context of health services refers to a lack of uptake and poor adherence (i.e. continued use) to an intervention. For example, a study evaluating a self-directed Internet-based mental health intervention (MoodGYM) in high schools found that only 8.5% (45/527) of participants logged on to use MoodGYM, and very few proceeded beyond the first part of the programme [24]. The importance of engagement with DHIs cannot be overstated, as research suggests that greater adherence and engagement are generally associated with more positive clinical outcomes [25–27].

Several studies have found that engagement and adherence to an intervention may relate to certain characteristics to do with the intervention, the user, or the condition targeted. For example, reasons for poor engagement in online therapy have included participants finding the intervention too demanding and being unable to find time to complete tasks [28], preferring face-to-face therapy with a human therapist [29], and experiencing problems with their computer or poor Internet access [30]. One study evaluating youth engagement with an app for depression found participating in a monitored session significantly improved adherence with an average of about six more sessions being completed for those in the monitored group. In addition, other predictors of greater adherence were sex (being female), living in a rural area and lower pre-test anxiety [31].

For mental health apps in particular, reasons identified for low engagement include poor usability (i.e. difficult to use or unenjoyable content), lack of user-centric design (i.e. not meeting the needs of the user), concerns about privacy and trust, and the unhelpfulness of apps in emergencies [32]. Creating and maintaining interest for adolescents are of key importance when designing DHIs. Indeed, Ritterband et al. [33] argued the need for three main components (or what are often termed “essential ingredients”) to provide a more immersive and engaging environment: (i) multimedia (e.g. audio, visual, and image components), (ii) interactivity, and (iii) personalisation. Although these components were not specified for any age group in particular, adolescents tend to prefer audio, visual, and interactive programmes to keep them engaged [34]. In terms of personalisation, Ritterband et al.’s [33] suggestion is consistent with the literature in that several reviews demonstrate that tailoring leads to improved engagement and better outcomes in attitude, behavioural intention, and behaviour change [35], although there is a lack of evidence regarding the optimal form of personalised support in terms of DHIs [31].

User engagement appears to be crucial in successfully implementing digital interventions and for positive outcomes. Reduced adherence means patients do not benefit from the full effects of the treatment, which impacts on recovery and in turn leads to increased healthcare costs [36]. Behaviour change mechanisms within interventions are unlikely to have any effect if participants are only briefly exposed to them [37]. Moreover, much of the literature to date has been on factors affecting engagement and adherence for adult populations, and there is a paucity of studies in youth populations. More research needs to be conducted in this area, as factors that may affect adult populations may not necessarily relate to a youth population. As developers of DHIs need to understand what the essential components are to better engage users, more studies need to carry out rigorous evaluations to precisely determine these factors. Moreover, the use of human support with SPARX to improve adherence has never been trialled before. We will address this in our work by trialling a novel-supported and personalised version of SPARX to evaluate whether there is an added benefit of human support on adherence and engagement rates.

### Aims

This pilot and feasibility RCT aims to evaluate SPARX for adolescents with mild to moderate depression in England to inform the development of a future definitive RCT.

### Objectives

The feasibility objectives of the trial are to examine the use and recruitment of SPARX in particular settings, specifically Child and Adolescent Mental Health Services (CAMHS), school-based Mental Health Support Teams (MHST), general practitioner (GP) practices, approved previous trial cohort databases (i.e. National Institute for Health and Care Research/NIHR BioResource), and resources (i.e. MyHealthE). The feasibility objectives of the intervention are whether supported SPARX has an added benefit on adherence (i.e. how many levels in total participants completed) and engagement (i.e. overall response to, sense of immersion and satisfaction with the intervention). Feasibility of the intervention will be determined by the proportion of participants completing at least four modules of SPARX, as this is deemed to be the minimum effective dosage given that is where the main therapeutic content is delivered. More than 80% completion would be considered feasible, whereas below 40% will be considered not to be feasible. For the likely outcome of completion rates falling between these limits, detailed analysis of trial data, including process evaluation qualitative interviews, will be used to inform the approaches that may improve adherence rates in subsequent trials. Value added by the supported SPARX intervention will be considered in terms of how much greater, if at all, the adherence rates are. The pilot objective is to estimate the variance of change in the primary outcome measure within groups (Patient Health Questionnaire modified for adolescents [PHQ-A] [38]) to calculate an appropriate sample size for the full definitive trial.

## Methods

### Trial design

This trial is a single-blind, three-arm, pilot, and feasibility randomised controlled trial, with an embedded process evaluation. The research assistants who conduct the baseline and primary endpoint assessments are blinded. The study settings are CAMHS, MHSTs, GP practices, and two databases/resources (NIHR BioResource and MyHealthE). CAMHS are National Health Service (NHS) centres that assess and treat adolescents with emotional, behavioural, or mental health difficulties. MHSTs are a recent government initiative designed to support young people with mental health issues in education settings. GP practices are primary medical services in the UK that treat all common health conditions and refer patients to hospitals and other medical services for urgent or specialist treatment. The NIHR BioResource is a recallable resource of over 250,000 volunteers, with and without health conditions, who have agreed to take part in health-related research. MyHealthE is an online portal where parents and caregivers can complete routine outcome measures digitally, and as part of this system, parents can then give their permission to be contacted with invitations to take part in other research studies, that is, providing their consent to be contacted. All CAMHS, MHSTs, and GP practices will be based in England. We aim to recruit 120 participants (aged 11–19 years) in total. Participants will be followed up at weeks 4 and 8–10 after baseline. Participants will receive £20 worth of Amazon vouchers for each assessment time point completed.

Our protocol follows the Consolidated Standards of Reporting Trials (CONSORT) extension for randomised pilot and feasibility trials [39] and the Standard Protocol Items: Recommendations for Interventional Trials (SPIRIT) [40]. Please see Additional file 1 for our SPIRIT checklist.

This protocol has also embedded patient and public involvement (PPI) throughout its development, following recommendations from the NIHR and UK standards for public involvement (see: https://sites.google.com/nihr.ac.uk/pi-standards/home). More detail can be found below on how PPI has shaped this programme of work.

#### Recruitment procedure for CAMHS/MHST

Across all *CAMHS/MHST* sites (Patient Identification Centres [PICs] and research site), the procedure will be as follows: the initial approach will be from a member of the patient’s usual care team (i.e. CAMHS/MHST clinician/practitioner), and information about the trial will be given to potential participants. All individuals conducting initial patient identification at sites will be given the inclusion/exclusion criteria for the study. The usual care team will provide the adolescent and their parent/guardian a participant information sheet. Once the study has been discussed and information sheet given to the adolescent/parent/guardian, a member of the patient’s usual care team will send their contact details confidentially by email to the research team with the family’s consent. The parent/guardian participant information sheet will also have a QR code and website link to an online consent to contact form specifying their preferred mode of contact (e.g. telephone or Microsoft Teams). Parents/guardians who provide consent to contact will be approached by a member of the research team who will explain the study process and ascertain initial screening eligibility over the phone/Microsoft Teams to determine the presence of any obvious exclusion criteria. Consent will be obtained via videoconferencing to provide some assurance of identity. It is a requirement that parents/guardians consent to the trial as they will be completing the Development and Well-Being Assessment (DAWBA) [41] and other measures (side effects questionnaire). However, there will be a dual consent process for participation, with under 16 years old providing child assent along with their parent/guardian providing written consent. Those who are 16 years and over can provide their own written consent.

Members of the clinical care team, or clinical research study officers at study sites, will be asked to record the numbers of patients approached (and to record the reason for nonparticipation). For those who consent to contact, parents’/patient’s contact details will be provided to the research team. Reasons for nonparticipation will be recorded (where given) for CONSORT purposes.

#### Recruitment procedure from GP practices

For participants identified and recruited from GP practices, the procedure will be as follows: utilising an approved search with filters to identify potentially eligible participants, staff at GP practices will conduct a search of their database which will generate a list of potential participants. At the practice, a member of staff will use a letter/email/SMS template to send to families, which will include information about the study and a flyer with brief information about the trial or a participant information sheet. Parents/guardians will be able to provide written consent to contact via a QR code or website link to an online consent to contact form. Parent/guardians who provide consent to contact will be approached by a member of the research team who will explain the study process and ascertain some screening eligibility over the phone/Teams to determine the presence of any obvious exclusion criteria. Consent will be done via videoconferencing to provide some assurance of identity.

#### Recruitment procedure from MyHealthE

Another means of participant recruitment for the trial will be in using MyHealthE — an online portal for the automated screening of referred families using NHS CAMHS data. MyHealthE uses a secure text/email system through which primary caregivers are invited to register and complete validated clinical screening measures using an online portal. These are then automatically coded using standard algorithms to subsequently allow the research team to identify potentially eligible participants. MyHealthE seeks parents’ permission to be contacted with invitations to take part in research studies. By consenting to research contact, parents also give their permission for NHS trust-approved researchers to review their children’s medical records to establish eligibility. In some of the participating organisations, clinical triage and rapid screening for mild to moderate levels of anxiety and depression will occur as part of routine care using MyHealthE.

Members of the SPARX-UK research team will regularly log in to the MyHealthE researcher portal (this portal only includes information about children whose parents gave consent to contact) to check for any new cases flagged as “eligible”. Researchers will identify potential participants by reviewing cases flagged up as “eligible” by MyHealthE using a standard algorithm. The “eligible” flag will be treated as a potential participating family, who the research team will then contact with an invitation to take part in the study.

#### Recruitment procedure from NIHR BioResource

The NIHR BioResource is a recallable resource of over 250,000 volunteers, who have agreed to take part in health-related research. We will be approaching selected potential participants from two trials within the NIHR BioResource: Genetics Links to Anxiety and Depression (GLAD) and the DNA, Children + Young People’s Health Resource (D-CYPHR). Participants in the GLAD trial have been referred through clinics or self-referral, with all participants having mild to moderate anxiety and depression. GLAD participants are all 16 years and over, whilst the D-CYPHR trial participants are 0–16 years. NIHR BioResource will create a list of potential participants based on our inclusion/exclusion criteria, which is then input into a secure database. An automated message is sent to selected parents. Once a parent expresses an interest in taking part by completing consent to contact, members of the research team will make contact to arrange an initial screening appointment.

#### Screening

After the research team receive consent to contact and the associated contact details, they will arrange a telephone/videoconferencing screening appointment with the parent/guardian or adolescent (if they are 16 years and over). This screening approach has shown to be effective in previous trials of similar interventions [42, 43]. At this point, the following details will be recorded: contact details, age of depression onset and brief clinical history, previous contact with healthcare services, previous/current medications or therapy for depression, access to Internet/PC/Mac/laptop/smartphone, other diagnoses, and inclusion/exclusion criteria. The researcher will outline the time commitment involved in the trial at this stage. Adolescents meeting any exclusion criteria or not meeting all inclusion criteria at this time point will not be invited to the baseline assessment. If the adolescent is not eligible for the study or does not wish to attend a baseline appointment, the researcher will record the reason for nonparticipation. Reasons for not attending a baseline assessment will be recorded for CONSORT reporting.

#### Baseline and consent appointment

Families who meet the initial eligibility screening criteria and who are willing to attend a Microsoft Teams baseline appointment will be asked to complete a DAWBA [41] assessment online prior to the baseline appointment. Only the parent/guardian will complete the DAWBA.

Adolescents and one of their parents/guardians will be invited to attend a remote baseline appointment via Microsoft Teams. At this appointment, the details collected over the telephone/videoconference for the patient will be checked. The researcher will complete the consent process with the adolescent and their parent/guardian, and they will be consented into the trial. During this assessment, the researcher will send a live link to participants on Microsoft Teams where the participant will have an opportunity to read the information sheet again and then complete the consent form. The researcher will complete a paper or online version of an intellectual disability screening measure (Child and Adolescent Intellectual Disability Screening Questionnaire [CAIDS-Q]) [44] and the PHQ-A with adolescents to confirm eligibility. The researcher will then complete paper or online versions of all baseline measures with eligible participants. The participant will be given their study ID at the point of randomisation, at which point they are enrolled into the study. A log will be kept matching screening identification (IDs) with study IDs. Randomisation will occur via REDCap at or just after the baseline appointment (i.e. within 2 weeks), prior to starting the trial. The method of randomisation is block randomisation, performed independently of method of referral.

#### Additional eligibility assessments will be undertaken for those recruited from GP practices, NIHR Bio-Resource, and MyHealthE

As these sources will not have a suitably qualified NHS healthcare professional to confirm the presence of mild to moderate depressive symptoms, the chief investigator (CI), who is a consultant child and adolescent psychiatrist, will review screening and assessment data to confirm eligibility. If eligible, randomisation will be confirmed by the research assistant. This eligibility decision will be made within 2 weeks of the information being presented. A log will be kept recording the assessment outcome.

#### Ethical approval

The SPARX-UK trial obtained ethical approval from the South West-Cornwall & Plymouth Research Ethics Committee on 15 December 2022 (Ethics Ref.: 22/SW/0149) and has been registered with ISRCTN, trial number 15124804.

**Fig. 1:**
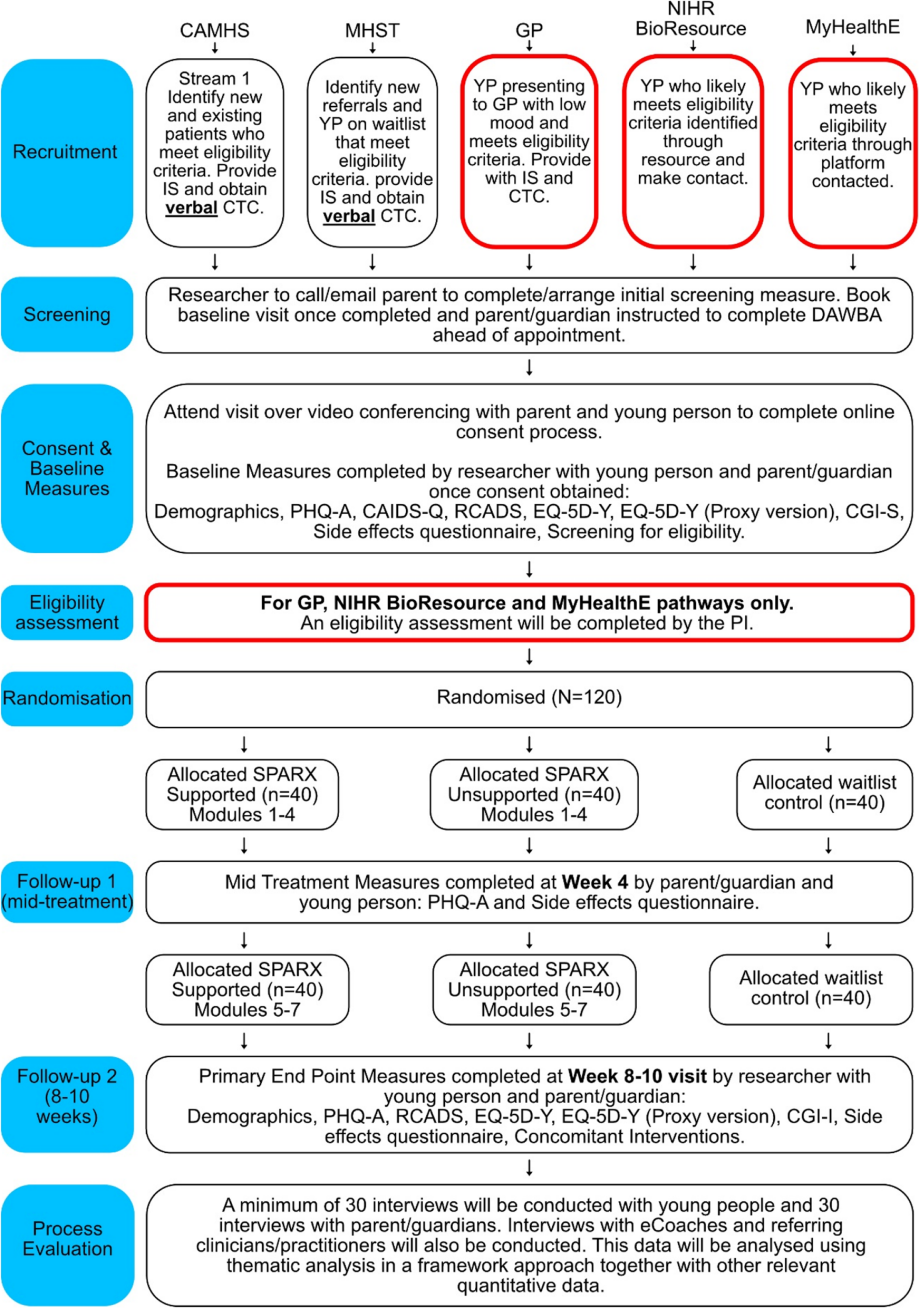
Schematic diagram of trial design. Note: CAMHS, Child and Adolescent Mental Health Services; MHST, Mental Health Support Teams; GP, general practitioner; NIHR, National Institute for Health and Care Research; IS, information sheet; CTC, consent to contact; DAWBA, Development and Wellbeing Assessment; CAIDS-Q, Child and Adolescent Intellectual Disability Screening Questionnaire; CGI-S/I, Clinical Global Impressions Severity/Improvement; PHQ-A, Patient Health Questionnaire modified for adolescents; RCADS, Revised Child Anxiety and Depression Scale

A schematic diagram of the trial design is shown in Fig. 1.

### Study participants and sample size rationale

Participants in this trial are the adolescents, and it is also a requirement that one of their parents/guardians are also consented to the trial. One of the aims of this study is to estimate the variance of the primary outcome measure (change in PHQ-A), so that a sample size can be computed for a subsequent trial of intervention efficacy. There are multiple methods suggested for sample sizes in pilot studies, but here the numbers are reflecting the smallest sample size (pilot plus subsequent efficacy trial), for which 40 per arm is reasonable [45]. A sample size of 40 per arm should be sufficient to avoid under or overpowering the main study whilst not making the pilot study excessively large.

#### Study population

Study participants will be adolescents aged 11 to 19 years with mild to moderate levels of depression.

#### Eligibility criteria

##### Inclusion criteria

Adolescents will be eligible for the study if as follows:

- They are aged 11 to 19 years on the date of consent (researcher confirms with parent/guardian at screening).
- Identified as having symptoms of mild to moderate depressive disorder
- Able to provide written consent or, if under age 16, written parental/guardian consent and written/verbal child assent (confirmed at baseline)
- Have access to a computer with Internet access or smartphone or device to use SPARX and must be able to install and log in (confirmed at baseline)
- They and their parent/guardian can read and write in English (confirmed at baseline).

##### Exclusion criteria

Adolescents will not be able to enter the study if ANY of the following apply:

- Clinical concerns that depression is too severe to benefit from SPARX and self-harm/suicidal risk is too high
- Intellectual disability or physical limitations precluding the use of SPARX (confirmed at baseline via CAIDS-Q)
- Had (in past 3 months) or currently having treatment with CBT/interpersonal therapy (confirmed at baseline)
- Has another major mental health disorder (e.g. psychosis, eating disorder) where the primary focus was not depression as confirmed by a clinician or DAWBA
- Safeguarding concerns that are not currently being managed (i.e. the adolescent is the subject of a safeguarding investigation) as confirmed by a clinician

### Trial setting

This trial will be run online across England. Our main research sites are Oxford Health NHS Foundation Trust and Nottinghamshire Healthcare NHS Foundation Trust with multiple PICs covering several regions of England. Adolescents will be followed up at weeks 4 and 8–10 weeks after baseline.

### SPARX intervention

SPARX is a DHI for young people with mild to moderate depression and was designed as a stand-alone self-help intervention that can be accessed online via a computer or mobile phone/tablet app. SPARX was created to address the gap in adolescent depression treatment options, and it uses CBT techniques to address symptoms of depression. SPARX uses elements of fantasy gaming to engage the user in experiential learning with a Guide acting as a “virtual therapist” to explain how to use the skills learnt in the gaming environment into “real life”.

At the beginning and end of each module, the user interacts, in the first person, with a character called the “Guide”, who provides psychoeducation, gauges mood, and sets and monitors real-life challenges, equivalent to homework. The user is then transported to the “game world” to undertake interactive challenges. Upon successfully completing each module, the user returns to the Guide who puts the skills learnt in the game world into a ‘real-life’ context and sets a ‘challenge’ (i.e. CBT homework task) to facilitate skill generalisation. SPARX uses evidence-based CBT skills that focus on six ‘gems of power’ specifically: “Relax” (relaxation training), “do it” (e.g. behavioural activation), “sort it” (e.g. social skills training), “spot it” (recognising or naming cognitive distortions), “solve it” (problem-solving), and “swap it” (e.g. cognitive restructuring).

There are seven modules (or levels) in total, and each one lasts approximately 30 min. A breakdown of the core content of each module is outlined in Table 1.

**Table 1:**
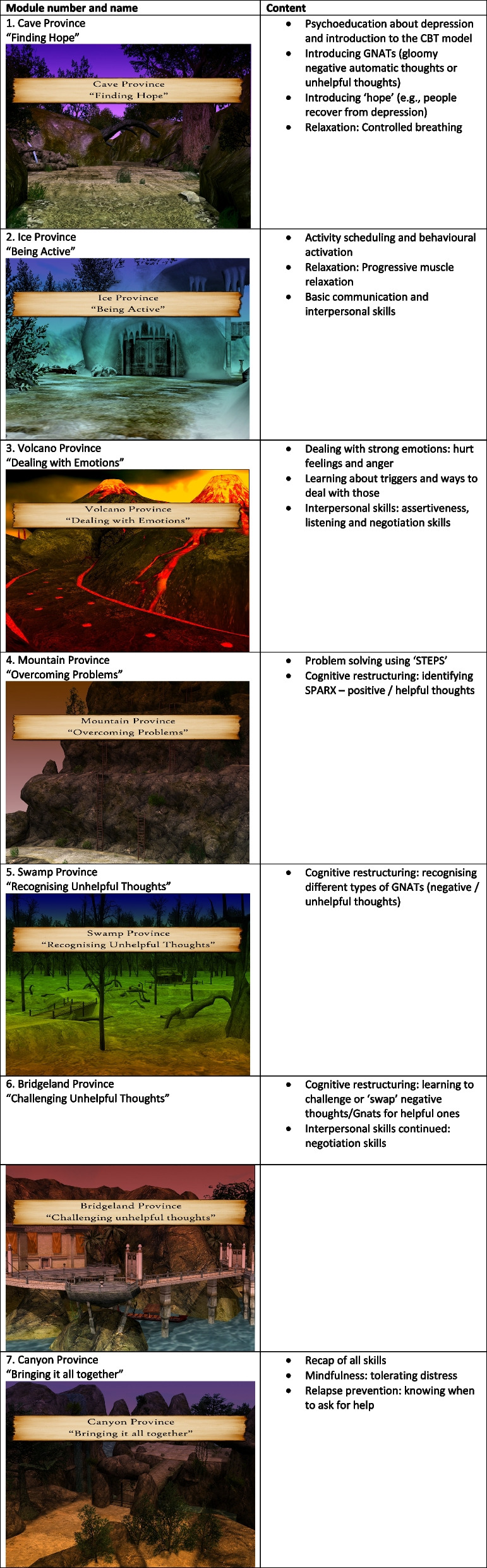
Core content in the seven modules within SPARX

After consulting with various stakeholders, the version of SPARX being used for this trial will retain the voice actors from the original New Zealand version. However, there will be differences from previous studies and the version available to the New Zealand public, including the following:

- No in-app measures or associated feedback
- The added ability to replay previously completed modules
- Localisation of specific guidance (e.g. signposting to UK-based not New Zealand-based support services)

### Human support

Participants randomised to receive supported SPARX will receive personalised guidance from an e-coach throughout their use of the intervention. The e-coach will provide limited contacts (less than 15 min per module) according to our study’s e-coach manual, as well as adherence reminders in case a module is not completed within 7 days. The feedback to be provided has the primary purpose of supporting, encouraging, and motivating participants, as well as promoting adherence to the intervention. In the supported arm, e-coaches will provide personalisation options to participants. This will include a choice of one or two modules being released per week and the contact modality (e.g. MS Teams/phone/email). E-coaches will also be able to access basic SPARX usage data for the participant(s) they are supporting throughout the trial. The e-coaches are psychology assistants, who are graduates in psychology and all work within CAMHS. They will be supervised by a Professor of Child and Family Mental Health with extensive clinical expertise working with adolescents (PS) and a researcher with a clinical background and extensive knowledge of SPARX (ML). The use of support whilst using SPARX has not been explored in any previous trials.

### Waitlist control group

The control group is a waitlist group. They will mostly be on waiting lists for services and will not have access to the SPARX intervention; however, they may receive support from other sources which we will capture at the primary endpoint via the concomitant interventions measure. Adolescents will receive £20 worth of Amazon vouchers for each assessment time point completed.

### Outcomes

#### Primary outcome measures

*Feasibility measures*: This includes feasibility of the intervention and feasibility of the trial measures. These data will be collected throughout the trial by researchers and used to inform the feasibility of conducting a future RCT.

*Feasibility of intervention measures*: This includes adherence to the intervention and qualitative information on acceptability and feasibility.

*Feasibility of trial measures*: This will consist of numbers approached/numbers consented and randomised, errors in randomisation, drop-out rates at each time point, outcome measure completion rates, instances of unblinding, and protocol deviations.

*Pilot outcome*: The variance of the change in *Patient Health Questionnaire modified for adolescents (PHQ-A)* [38] from baseline. PHQ-A is adapted from the PHQ-9 and modified for adolescents. It is a 9-item measure with each measure rated on a 4-point scale that assesses the severity of depressive disorders and episodes) in children and adolescents. The measure is completed by the adolescent. Each item asks the adolescent to rate the severity of their depression symptoms during the past 2 weeks. The total score can range from 0 to 27, with higher scores indicating greater severity of depression. There are four additional items which relate to difficulties performing everyday tasks, suicidal thoughts, and attempts; however, these are not scored. The questionnaire has established validity and reliability [38]. A 5-point change is viewed as clinically significant [46]. The PHQ-A was chosen as the primary outcome measure, as this was the preferred option of our patient and public involvement (PPI) group over other commonly used measures of depression.

#### Other outcome measures

*Revised Child Anxiety and Depression Scale (RCADS)* [47]: The RCADS is a 47-item self-report questionnaire that measures symptoms of depression and anxiety in children and adolescents. The RCADS consists of six subscales helpful in screening children and adolescents for high prevalence disorders, including separation anxiety disorder, social phobia, generalised anxiety disorder, panic disorder, obsessive compulsive disorder (OCD), and major depressive disorder. A Total Anxiety Scale score (sum of the five anxiety subscales) and a Total Internalizing Scale score (sum of all six subscales) are derived, with higher scores indicating increased symptom severity. The RCADS has demonstrated good structural validity, reliability, and convergent and discriminant validity [47]. The RCADS is a widely used instrument within CAMHS and MHSTs.

*EQ-5D-Y and EQ-5D-Y (Proxy version)* [48, 49]: The child-friendly EQ-5D version (EQ-5D-Y) was introduced by the EuroQol Group in 2009 as a more comprehensible instrument suitable for children and adolescents and is based on the EQ-5D-3L. The EQ-5D-Y comprises the following five dimensions: mobility, looking after myself, doing usual activities, having pain or discomfort, and feeling worried, sad, or unhappy. Each dimension has three levels: no problems, some problems, and a lot of problems. The adolescent is asked to indicate his/her health state by selecting the most appropriate statement in each of the five dimensions. This decision results in a 1-digit number that expresses the level selected for that dimension. The digits for the five dimensions can be combined into a 5-digit number that describes the adolescent’s health state. The EQ VAS records the adolescent’s self-rated health on a vertical visual analogue scale where the endpoints are labelled “The best health you can imagine” and “The worst health you can imagine”. The EQ-5D-Y (Proxy version) asks the caregiver (the proxy) is to rate the child’s/adolescent’s health-related quality of life in their (the proxy’s) opinion. Both measures have established validity and reliability [48, 49] and will be used in this study to measure health related quality of life.

*Clinical Global Impressions Scale (CGI)-Severity/Improvement* [50]: The CGI provides an overall clinician-determined summary measure that considers all available information, including a knowledge of the patient’s history, psychosocial circumstances, symptoms, behaviour, and the impact of the symptoms on the patient’s ability to function. The CGI comprises two companion one-item measures evaluating the following: (a) severity of psychopathology from 1 to 7 and (b) change from the initiation of intervention on a similar 7-point scale. The CGI-S asks one question: “Considering your total clinical experience with this particular population, how mentally ill is the patient at this time?” which is rated on a 7-point scale (from 1 = normal, not at all ill to 7 = amongst the most extremely ill patients). The CGI-I consists of one item: “Compared to the patient’s condition at admission to the project this patient’s condition is…” and is rated from 1 = very much improved since the initiation of intervention to 7 = very much worse since the initiation of intervention. The questionnaire has established validity and reliability and is widely used in clinical research [51].

*Concomitant interventions*: To assess what other mental health treatments/interventions the adolescent is accessing during the study period, parents/guardians will be asked to complete a short questionnaire, which asks about current diagnoses and treatments/interventions/medication in progress. This will be completed at baseline as part of the demographic questionnaire completed with the researcher and then again at 8–10-week follow-up with the researcher via telephone/Microsoft Teams.

*Usage data*: Indices such as number of modules completed, time spent using SPARX, and number of logins will be collected via an in-built SPARX feature, and time spent with e-coaches (if in supported arm) will be recorded in REDCap. These data will be collected throughout the trial and will be used to measure adherence and engagement as part of the process evaluation.

#### Adverse events

Adverse events/side effects will be recorded by the PHQ-A and on a modified version of the side effects scale developed by Hill and Taylor [52]. An adverse event will be recorded where there is clinically meaningful deterioration from baseline on the PHQ-A. On the PHQ-A, a > = 5-point increase (i.e. moving one severity category) is regarded as clinically meaningful [46], and thus, an adverse event will be recorded when this occurs or where there is an increase of one, or more, point from baseline to follow-up (4 weeks and/or 8–10 weeks) on item 9 or a positive response (i.e. “yes”) on any of the two additional suicide items from baseline to follow-up. The Hill and Taylor [52] scale consists of 17 short items relating to common side effects (such as headaches, anxiety, sleep, and low mood). The participant is asked to respond on a 5-point scale ranging from “not at all” to “all the time” to describe the presence of each item. A score on any item that is equal to or greater than 2 (“about half the time”) and greater than their baseline score is recorded as an adverse event on this scale. All adverse events/side effects will be captured at baseline (to ascertain presence of these symptoms prior to the intervention), mid-intervention, and 8–10-week follow-up.

#### Screening measures

*Development and Wellbeing Assessment (DAWBA)* [41]: The DAWBA is a package of interviews and questionnaires completed by parents/guardians only to reduce the assessment burden on the adolescent. This approach has been commonly taken in other trials with children and young people and their parents [42, 53]. The DAWBA is designed to generate ICD-10 and DSM-IV/DSM-5 psychiatric diagnoses for children and young people. The DAWBA computer algorithm estimates the probability of having a psychiatric disorder in bands of <. 1%, 0.5%, 3%, 15%, 50%, and > 70% based on large community-based population studies [41], and the top two levels have been shown to reliably indicate the presence of a clinician-rated diagnosis and can be used as an alternative to clinician-rated diagnoses in research studies [54]. The DAWBA has established validity and reliability [41]. The parent-reported DAWBA starts with a Strengths and Difficulties Questionnaire (SDQ) [55]. If the participant is later enrolled in the trial, this will be used as their baseline SDQ. DAWBAs that score people as being likely to have any conditions will be second reviewed by the chief investigator (a medical practitioner) to ascertain that they should be excluded from the trial. It must be noted that the DAWBA is not being used in this trial to diagnose depression but to establish possible comorbid disorders and determine study eligibility (i.e. whether to exclude potential participants from the study).

*Child and Adolescent Intellectual Disability Screening Questionnaire (CAIDS-Q)* [44]: The CAIDS-Q is being utilised to determine the presence of intellectual disability at baseline. The questionnaire contains seven items (two on literacy, one on telling the time, one on friendships, two on previous contact with specialist services and current educational support, and one on tying laces). They are answered in a yes/no format by someone who knows the person well, and some items can be tested directly with the adolescent (depending on age/communication). A total score is calculated which is converted to a percentage score. A cutoff (by age group) indicates if the child is likely to have an intellectual disability or not. The questionnaire has established validity and reliability [56].

#### Process evaluation

The process evaluation will follow the Medical Research Council’s (MRC) guidelines for evaluating complex interventions [57]. It will explore the components suggested in MRC guidelines, namely reach, dose, and fidelity of implementation of intervention, and make recommendations for adaptations for the future RCT. It will also examine the contextual factors and potential mechanisms underlying participant behaviour change and probe for any unexpected consequences.

Interviews will be conducted with adolescents assigned to either version of SPARX (target *n* ≥ 30) and parents/guardians of adolescents (*n* ≥ 30) after they have completed their 8–10-week follow-up. This sample size will be determined based on the model of information power where we will aim to achieve both breadth and depth of views [58]. Purposive sampling will be used so that a diverse range of views are voiced, including diversity in terms of ethnicity, socioeconomic status, and adherence to the intervention (i.e. interviewing those who completed 0 level to treatment completers). Interviews with e-coaches supporting the intervention (target *n* ≥ 3) and clinicians recruiting to the study (*n* ≥ 5) will be conducted during trial delivery. Online feedback from participants will also be analysed together with usage indices recorded as part of the online system such as total time spent with e-coach, number of modules completed, and number of logins. A brief online questionnaire will be given to participants who drop out of the trial early to gain a more holistic overview of engagement with SPARX. A full process evaluation protocol will be published.

Table 2 outlines the completion time points for each outcome measure and who completes them.

**Table 2:** Baseline and outcome measures

Months post-randomisation	0	0	1	2
Time point	Telephone/videoconference screening	Baseline	Mid-intervention (4 weeks)	Primary end point (8–10 weeks)
Consent		R (P+A)		
Randomisation		R		
PHQ-A		R (A/P)	A/P	R (A/P)
CAIDS-Q		R (A/P)		
RCADS		R (A/P)		R (A/P)
Screening for eligibility	R (P)	R		
SDQ & DAWBA (conducted post telephone/videoconference screen & prior to baseline)	P			
CGI-S/I		R (A/P)		R (A/P)
EQ-5D-Y		R (A)		R (A)
EQ-5D-Y (proxy version)		R (P)		R (P)
Demographics		R (A/P)		
Concomitant interventions		R (A/P)*		R (A/P)
Adverse events		R (A/P)	A/P	R (A/P)
Interview (process evaluation)				R

*P* parent: *A* adolescent: *R* researcher: *PHQ-A* Patient Health Questionnaire modified for adolescents: *CAIDS-Q* Child and Adolescent Intellectual Disability Screening Questionnaire: *RCADS* Revised Child Anxiety and Depression Scale: *SDQ* Strengths and Difficulties Questionnaire: *DAWBA* Development and Wellbeing Assessment: *CGI-S/I* Clinical Global Impressions Severity/Improvement.*Taken as part of demographic questionnaire at baseline

#### Safeguarding and participant care

No significant risks to physical safety are anticipated; however, changes in depressive symptoms may occur in adolescents during the trial and cause psychological harm. Although a formal data monitoring committee is not required due to the minimal physical risks associated with the intervention, we will have an independent scientific advisory board, which serves a similar function to a Trial Steering Committee (TSC), throughout the trial. To ensure participant safety, adverse events will be recorded at baseline, 4 weeks, and 8–10 weeks. Any related or unexpected serious adverse events will be reported to the research ethics committee.

#### Patient and public involvement

The current project sits within a programme of work with its own PPI group of young people with lived experience of accessing services for depression/mental health conditions, called Sprouting Minds. The initial study grant was reviewed and received feedback from Sprouting Minds. Since January 2022, a subset of Sprouting Minds have met regularly to advise and guide the research. The group comprises one parent and three young people with lived experience relevant to the study. PPI has shaped the design of the trial and its dissemination in the following ways:

- Reviewing and selecting outcome measures, including choosing our primary outcome measure (PHQ-A)
- Co-development of study materials including participant information sheets, the parent manual, and instructions on downloading SPARX
- Involvement in discussions regarding trial and recruitment processes
- Completing the intervention and providing feedback on content and all modules
- Attending ongoing meetings to provide PPI perspective and assist with troubleshooting (e.g. recruitment and retention)
- Guiding interview topics and shaping questions for the process evaluation interview schedules
- Providing feedback on signposting services and the design for accessing signposting within the intervention

The PPI group mainly contributes remotely to enable involvement from members who are not geographically close, and all members are paid for their time in line with National Institute for Health and Care Research (NIHR) involvement guidelines. Their advice and input will be sought throughout the trial including participation in facilitating and analysis of the interviews with adolescents and parents/guardians, co-creating lay summaries, and other dissemination materials including involvement in conference presentations and as co-authors on publications.

### Statistical methods

#### Quantitative analyses

Summary statistics will be presented for this trial using Microsoft Excel. No interim analyses are planned. Summary statistics, proportions, and standard deviations will be given to estimate the change in PHQ-A, variance, and recruitment rates (per month) in different settings and whether there is compliance (defined as the percentage completing four levels or more of SPARX). The primary pilot outcome is the variance in change in the primary outcome measure (PHQ-A). We will not stratify at this point because we are estimating variance, not effect, and we are assuming they are similar, although this will be checked. A full analysis plan will be made available before unblinding of data, and anything that is not planned will be considered data dependent and considered speculative [59].

#### Qualitative analyses

In-depth semi-structured qualitative interviews with adolescent participants receiving the online intervention and their parent/guardian will capture general feedback on their experiences of taking part in the trial as well as satisfaction and acceptability of the intervention as part of the process evaluation. We will explore, with both participants receiving the intervention and intervention supporters (i.e. the e-coaches), their experiences of receiving and supporting the intervention online, mechanisms of impact, barriers, and facilitators to taking part in the intervention and in their continued involvement. Referring clinicians and e-coaches will also be interviewed to gain their experiences of being involved in the trial.

All interviews will be audio-recorded and transcribed by a University of Nottingham-approved external transcription company or by a member of the research team. Transcripts will be anonymised before analysis. Interviews will be analysed using reflexive thematic analysis [60], and, more broadly, themes will be organised using the framework method [61]. All analysis will be conducted using NVivo 12. Coding and organisation of codes will be cross-checked within the research team to ensure validity.

## Discussion

This study is the first of its kind to investigate an online serious gaming intervention called SPARX for adolescents with mild to moderate depression in the UK and to explore whether there is better uptake with supported versus self-directed completion of SPARX. By offering an intervention remotely to adolescents with depression in England, we hope to offer an alternative intervention based on CBT principles for adolescents that can become mainstream and thus improving provision of care within the health service. Given the complexities and barriers with which adolescents with depression can present and face, including not being able to access treatment or avoiding face-to-face interventions due to stigma, online interventions are an especially important treatment pathway to consider going forward. With cCBT recommended in NICE guidelines for the treatment of adolescents with mild depression, this trial is especially important. Furthermore, by refining our recruitment process, data collection procedures, and running of the intervention itself, from this trial, we aim to prepare for a much larger subsequent RCT in which we will compare outcomes between our SPARX intervention (either supported or self-directed) with care as usual. Should the subsequent SPARX definitive trial prove effective, it will pave the way for a new and accessible type of intervention for adolescents across the UK.

### Supplementary Information

**Additional file 1.** SPIRIT 2013 Checklist: Recommended items to address in a clinical trial protocol and related documents.

## Data Availability

Not applicable. The manuscript does not contain any data.

## Abbreviations

CAIDS-Q: Child and Adolescent Intellectual Disability Screening Questionnaire
CAMHS: Child and Adolescent Mental Health Services
cCBT: Computerised Cognitive Behavioural Therapy
CGI-S/I: Clinical Global Impressions-Severity/Improvement scale
DAWBA: Development and Wellbeing Assessment
DHI: Digital health intervention
GNATs: Gloomy negative automatic thoughts
MHST: Mental Health Support Team
NICE: National Institute for Health and Care Excellence
NHS: National Health Service
PHQ-A: Patient Health Questionnaire modified for adolescents
PIC: Patient Identification Centre
PPI: Patient and public involvement
RCADS: Revised Child Anxiety and Depression Scale
RCT: Randomised controlled trial
SPARX: Smart, Positive, Active, Realistic, X-factor thoughts
TAU: Treatment as usual
